# Establishing an *in vivo *model of canine prostate carcinoma using the new cell line CT1258

**DOI:** 10.1186/1471-2407-8-240

**Published:** 2008-08-15

**Authors:** Melani AM Fork, Hugo Murua Escobar, Jan T Soller, Katharina A Sterenczak, Saskia Willenbrock, Susanne Winkler, Martina Dorsch, Nicola Reimann-Berg, Hans J Hedrich, Jörn Bullerdiek, Ingo Nolte

**Affiliations:** 1 Small Animal Clinic, University of Veterinary Medicine Hanover, Hanover, Germany; 2 Center for Human Genetics, University of Bremen, Bremen, Germany; 3 Institute of Laboratory Animal Science, Hanover Medical School, Hanover, Germany

## Abstract

**Background:**

Prostate cancer is a frequent finding in man. In dogs, malignant disease of the prostate is also of clinical relevance, although it is a less common diagnosis. Even though there are numerous differences in origin and development of the disease, man and dog share many similarities in the pathological presentation. For this reason, the dog might be a useful animal model for prostate malignancies in man.

Although prostate cancer is of great importance in veterinary medicine as well as in comparative medicine, there are only few cell lines available. Thus, it was the aim of the present study to determine whether the formerly established prostate carcinoma cell line CT1258 is a suitable tool for *in vivo *testing, and to distinguish the growth pattern of the induced tumours.

**Methods:**

For characterisation of the *in vivo *behaviour of the *in vitro *established canine prostate carcinoma cell line CT1258, cells were inoculated in 19 NOD.CB17-*Prkdc*^*Scid*^/J (in the following: NOD-Scid) mice, either subcutaneously or intraperitoneally. After sacrifice, the obtained specimens were examined histologically and compared to the pattern of the original tumour in the donor.

Cytogenetic investigation was performed.

**Results:**

The cell line CT 1258 not only showed to be highly tumourigenic after subcutaneous as well as intraperitoneal inoculation, but also mimicked the behaviour of the original tumour.

**Conclusion:**

Tumours induced by inoculation of the cell line CT1258 resemble the situation in naturally occurring prostate carcinoma in the dog, and thus could be used as *in vivo *model for future studies.

## Background

Only few species are known to spontaneously develop prostatic neoplasia; therefore the search for a suitable animal model for this disease is difficult. Currently, the dog is used as *in vivo *model for prostate malignancies in man, since it shows a similar metastatic pattern as well as age dependent development of malignant prostatic lesions [1-3]. Whereas prostate cancer is a frequent finding in man and even one of the leading causes of death in the Western world, it is less common in the dog. The prevalence is only 0. 2%–0. 6% [20]. Since this number is based on necropsy findings, the true number might be higher [4,5]. Although the relative number appears to be quite low, it results in an absolute count of estimated 60,000–180,000 affected dogs in the USA, 6,000–18,000 dogs in the UK, and 5,300–15,900 dogs in Germany, based on population.

Although there are some reports observing a permissive or protective effect of androgens [6-10], androgens do not seem to have an influence on development, growth, or metastasis in prostate carcinoma in dogs. Other than in human medicine, the diagnosis is usually made at a very late state of the disease, since for dogs no screening tests are available as there are for man [13]. Therefore treatment options for prostate cancer in the dog are limited and mostly remain to be palliative.

Despite the rather high incidence of prostate carcinoma in man, there are only three cell lines of human prostate carcinoma and their sub lines available. Thus, it is not surprising that there are only occasional reports about cell lines of canine prostatic carcinoma [11-13]. Hence the purpose of the present study was to establish an animal model, using the recently described cell line CT1258, which has been derived from a spontaneous canine prostate carcinoma [14,15]. Special attention was directed towards comparison of the clinical behaviour of the induced tumour in mice to the spontaneous tumour in the donor, as well as histological comparison. A comparison of the Ki67 index was performed in order to be able to address a potential change in proliferation. The Ki67 Index has been described to be associated with a poor outcome in human prostate cancer [18].

## Methods

### Donor

The cell line was derived from a prostatic tumour of a Briard, 10 years, male intact, which had been presented at the Small Animal Clinic of the University of Veterinary Medicine Hanover, Germany. The dog had a two week history of dyschezia, gain of abdominal girth, polydipsia, and loss of appetite. Clinical examination revealed an undulating and strained abdomen. Abdominal radiographs showed a highly reduced perceptibility of detail. On abdominal ultrasound an enlarged prostate could be detected, which contained several cysts.

Explorative laparatomy was performed and about 3000 ml abdominal fluid were obtained; the prostate itself was highly enlarged and several miliary masses were found in the mesentery. There was no evidence of contact metastases to abdominal organs or distant metastases to the lung. Biopsies have been taken under general anaesthesia; the cytological diagnosis of a highly malignant adenocarcinoma of the prostate resulted in euthanasia ad tabulam. Prior to euthanasia, biopsies have been taken for further histological examination and for cell culture. Histological staining and immunohistochemical staining as well as cytogenetic analysis was performed as described below.

### Cell line

The cell culture conditions, as well as the characteristics of the canine prostate carcinoma cell line CT1258 have been described previously [15].

### Animals

This study involved 19 NOD-Scid mice (10 male, 9 female). All animals were bred and maintained in a protected environment at the Institute of Laboratory Animal Science of the Hanover Medical School. The mice were fed autoclaved food and water *ad libitum*, any manipulation was performed in a laminar flow hood. The animals were observed on a daily basis and sacrificed depending on the clinical condition and the size of detectable tumour, respectively. The tumours were allowed to grow up to a diameter of 10 mm. Additional criteria for euthanasia were ulceration of subcutaneous masses, a reduced level of activity of the mice, and the loss of appetite. The animals were humanely sacrificed by cervical dislocation after inhalation of > 70% carbon dioxide. The study was approved by the Lower Saxony State Office of Consumer Security and Food Safety (33-42502-05/950), the ethical approval was sought from the University of Veterinary Medicine Hanover.

### Inoculation of Cells

The cells were harvested from the culture flasks with 1 ml of TrypLE Express (Invitrogen, Karlsruhe, Germany) and incubated at room temperature for 1 minute. L-199 medium (Invitrogen, Karlsruhe, Germany) supplemented with 20% FCS was added and the cells were centrifuged at 350 g for 15 minutes. The cell pellet was washed twice with PBS. The cells were resuspended in 200 μl PBS; immediately before application the suspension was aspirated into Insulin-Syringes (BD Micro-Fine, BD, Heidelberg, Germany) and inoculated either subcutaneously into the left flank of the animals or intraperitoneally.

For subcutaneous inoculation, the animals received 1 × 10^6 ^cells. For intraperitoneal application, mice received either 1 × 10^5 ^or 5 × 10^5 ^cells. The 19 NOD-Scid mice were subdivided into two groups: One group consisted of four female and five male mice, the animals received 1 × 10^6^cells subcutaneously each. The second group consisted of five male and five female animals, cells were inoculated intraperitoneally. Four of them (2 male, 2 female) received 1 × 10^5 ^cells; six animals (3 male, 3 female) received 5 × 10^5 ^cells, respectively.

### Necropsy and histological staining

Necropsy was performed immediately after death had occurred. Lung, liver, spleen, kidneys, gonads, bowel, and detected masses were removed, fixed in 10% buffered formalin, paraffin embedded, sectioned, and stained with hematoxylin and eosin; immunohistochemical staining for Ki67 was performed, using the monoclonal antibody MIB-1 (Dianova, Hamburg, Germany) in a dilution of 1:100. The paraffin sections were pretreated in a microwave oven for 20 minutes in citrate buffer solution, pH 6. The secondary antibody was a biotinylated goat-α-mouse antibody, for detection, the Vectastain ABC-Kit (Vector Laboratories, Burlingame, USA) has been used. The Ki67 Index was determined by detecting the fraction of Ki67 positive stained cells in total 500 cells. This was performed in the original tumour, as well as in the induced tumours. Intestinal mucosa served as positive control. In addition, tumour samples of each mouse were obtained in liquid nitrogen for real time RT-PCR and in Hank's Medium for further *in vitro *culturing and Cytogenetic analysis. In order to address the growth pattern, the original canine tumour and the murine tumours have been classified based upon the presence of glandular, urothelial, squamoid, or sarcomatoid differentiation.

### Cytogenetic analysis

For cytogenetic investigation, cells have been processed as described previously [15]. Tissue was transferred to liquid nitrogen immediately after dissection and stored at -70°C until examination.

### Statistics

This study was intended to be descriptive rather than statistically significant. However, a paired student's t-test was performed. A P-value < 0.05 was considered to be significant.

## Results

### Pathological and histological examination

#### Original canine tumour

On histological examination the tumour showed to be consisting of poorly differentiated cells with numerous signs of malignancy. A strong anisocytosis, numerous cells with multiple nuclei, anisokaryosis, multiple mitotic figures, to some extent atypical mitotic figures, and a varying nucleus: plasma ratio could be detected. The tumour showed a compact growth with glandular differentiation. The Ki67 index was 43%.

#### Subcutaneous tumours

For analysis of duration until sacrifice of the mice, only those individuals were considered that were sacrificed due to tumour burden. All female mice and four of five male mice developed a detectable tumour mass after subcutaneous inoculation of 1 × 10^6 ^cells of the canine prostate carcinoma cell line CT1258 (Table 1). One mouse without detectable tumour growth was sacrificed due to bad clinical condition; necropsy revealed a mass of the thymus, histologically addressed as thymoma. In the remaining mice tumours were allowed to grow up to a size of 5–8 mm, which lasted 20 to 42 days (mean 25.37 days, median 22 days). Gender distribution showed a mean value of 28.75, median 26.5 days for female mice, and a mean and a median of 22 days for the males. None of the mice showed any signs of metastasis or of invasive growth pathologically or histologically; except for the mouse that did not develop any tumour mass, all animals remained in good clinical and nutritional condition.

**Table 1:** Tumour growth after subcutaneous injection of 1 × 10^6 ^cells of CT1258.

	Mean	No growth	Range	SD
	[days]	[No of animals]	[days]	
Male	22	1	20–24	1.63
Female	28.75	0	20–42	10.05

Total	25.38	1	20–42	7.58

**Figure 1:**
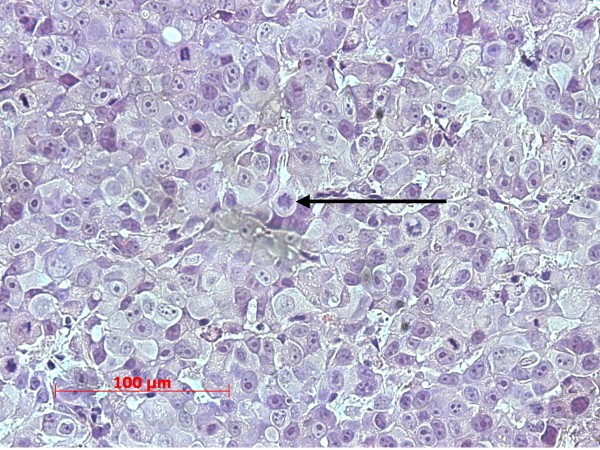
**Subcutaneous mass with a high mitotic index.** The arrow indicates an atypical mitotic figure.

**Figure 2:**
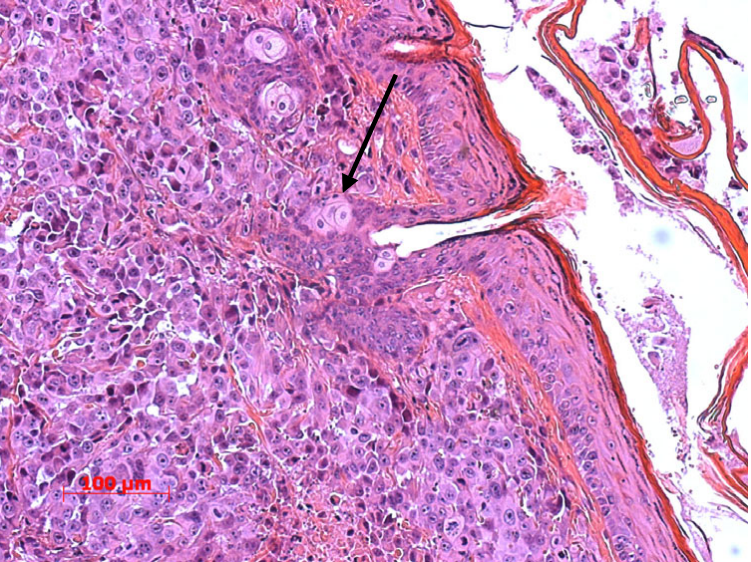
Subcutaneous mass with multiple mitotic figures and necrotic area; the large pale cells surrounding the hair follicle are part of the sebaceous gland and should not be mistaken as tumour cells (arrow).

**Figure 3:**
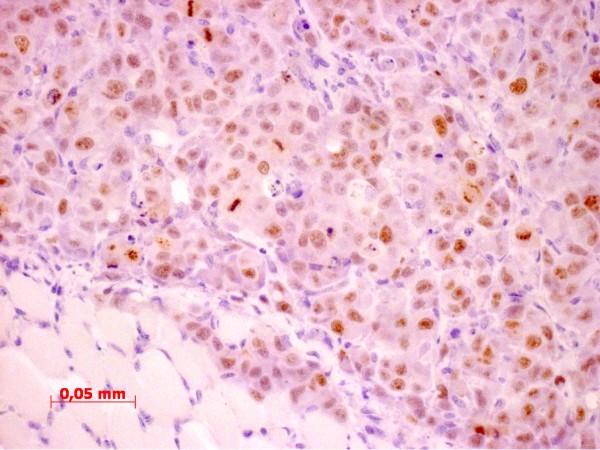
Ki67 staining of subcutaneous mass with a high amount of cells staining positively for Ki67.

Histology revealed a highly heterogeneous population of cells, numerous mitotic figures, anisokaryosis, anisocytosis, and several cells containing more than one nucleus; there was a variable nucleus: plasma ratio (Figures 1, 2). The centre of the obtained masses showed to be highly necrotic. Glandular differentiation was present. Immunohistochemistry revealed a strong positivity for the Ki67-antigen (Figure 3); the Ki67 index was between 41% and 48%, respectively

#### Intraperitoneal Tumours

The group was subdivided in two categories: Animals receiving 1 × 10^5 ^cells, consisting of two males and two females each, and animals receiving 5 × 10^5 ^cells, consisting of three males and three females each. The criteria for sacrification were impairment of the general condition, worsening of the nutritional condition, and/or reduction of activity level. Weight gain was not a reliable factor due to the development of massive peritoneal effusion.

**Figure 4:**
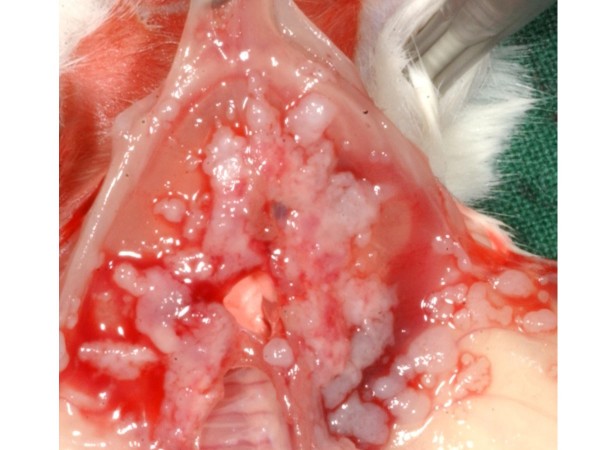
Diaphragm after i.p. injection of 5 × 10^5 ^cells with a high tumour burden; see Figure 5 for histology; the thoracic aspect of the diaphragm is not affected.

**Figure 5:**
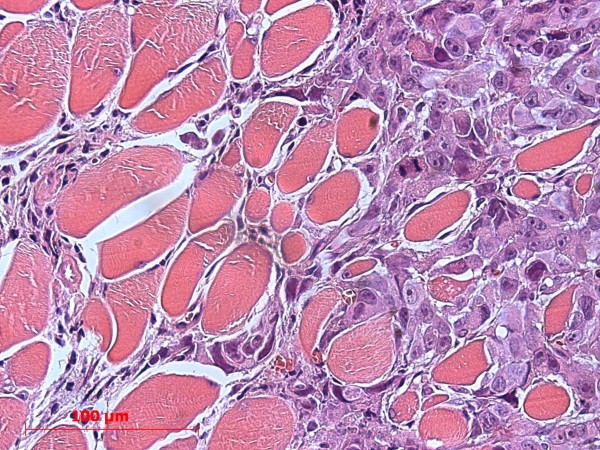
Diaphragmatic mass (see figure 4 for macroscopic appearance).

**Figure 6:**
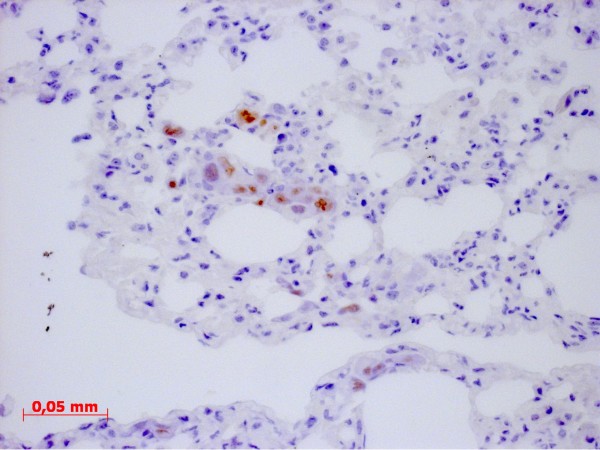
Ki67-staining of tumour cells in the lung; there is no evidence of vascularisation, therefore these cells are tumour emboli rather than metastasis.

In all mice receiving 1 × 10^5 ^cells tumour growth could be detected. The mean duration until sacrifice was 28 days. In one of three females and two of three males inoculated with 5 × 10^5 ^cells tumour growth could be detected, in this group the mean duration until sacrifice was 26 days (Table 2). Overall seven out of ten mice intraperitoneally inoculated with CT1258 developed tumour growth. Of those animals all but one male, which had received 5 × 10^5 ^cells, had an extended abdomen and proved to have peritoneal effusion. The male without obvious tumour growth had developed a thymoma, which was the cause for the bad clinical condition. Six of these ten mice developed a mass at the injection site, although at inoculation a proper amount of time had elapsed until withdrawal of the needle. Necropsy showed a moderate to high tumour burden at the peritoneum and no visible signs of metastasis to the lungs (Figure 4). Histological examination of the obtained masses showed a similar pattern as the subcutaneous masses with a glandular differentiation (Figure 5); staining for Ki67 was strongly positive with a Ki67 index between 45% and 47%. Isolated populations of tumour cells could be detected in the lungs, but there was no sign of vascularisation (Figure 6). In abdominal organs only peripheral tumour growth could be observed.

**Table 2:** Tumour growth after intraperitoneal injection of 1 × 10^5 ^and 5 × 10^5 ^cells.

	Mean	No growth	Range	SD
	[days]	[No of animals]	[days]	
Male	26.75	1	24–30	3.2
Female	27.67	2	24–30	3.21
1 × 10^5 ^cells	28	0	24–30	2.71
5 × 10^5^cells	26	3	24–30	3.46

Total	27.14	3	24–30	1.12

#### Cytogenetic Analysis

**Figure 7:**
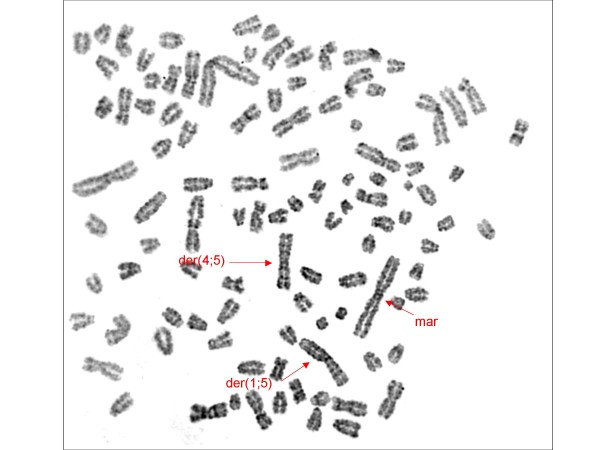
**Metaphase spread from cells derived from the original canine tumour.** The arrows indicate the derivative chromosomes der (1; 5), der (4; 5) and the marker chromosome mar, which consists of chromosome 1 and chromosome 2 material.

**Figure 8:**
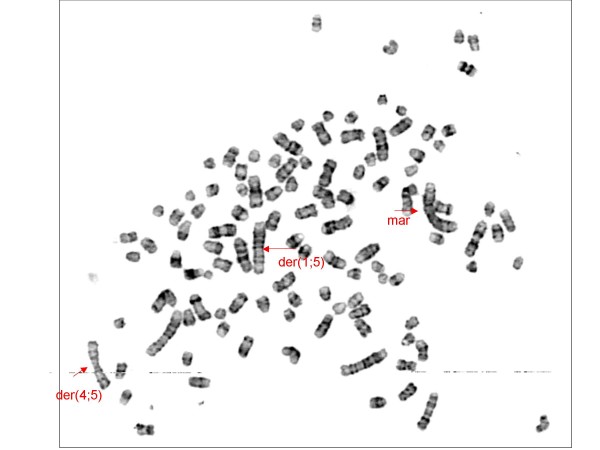
**Metaphase spread from cells derived from a CT1258 induced tumour.** The arrows indicate the derivative chromosomes der (1; 5), der (4; 5) and the marker chromosome mar, which consists of chromosome 1 and chromosome 2 material.

The cytogenetic investigation of intraperitoneal and subcutaneous tumours revealed the same markers that have been shown in analysis of the original tumour and of the cell line [15]. A hyperdiploid karyotype was present. Centromeric fusion between chromosomes 1 and 5 (der (1; 5)) and chromosomes 4 and 5 (der (4; 5)) were detected. A large biarmed marker (mar) was found (Figure 7, 8).

## Discussion

Ideally a cell line should not only be immortalized spontaneously but also be tumourigenic in experimental animals. Another claim may be the mimicking of the original natural behaviour of the tumour in the host. This condition promotes the predictability of any results obtained with that very cell line. This is true especially for cell lines derived from nonhuman tumours if the origin may serve as a model itself.

This study reveals that the cell line CT1258 is highly tumourigenic in NOD-Scid mice. Local tumour growth occurred in 89% of the animals that had received cells subcutaneously; 86% of the animals that had been inoculated intraperitoneally and that had developed tumour growth also developed local tumour growth at the injection site. The latter was not desired, and since it is not known how many cells remained in the branch canal, the size of those accidental masses cannot be compared accurately. Focussing on the mice with cells inoculated i.p., 86% developed peritoneal effusion just as the donor did and all of them had multiple small nodules within the serous membranes of the peritoneum rather than one large mass. The excellent local tumour growth on one hand and the rapid development of pleural effusion on the other hand indicates that in studies to come the use of the cell line might be of a greater value in local administration rather than in systemic inoculation. Orthotopic implantation might be of great value, since this could enable the assessment of local growth and invasion as well as potential metastasis. Due to the lack of vascularisation, the tumour cells found in the lungs of the mice must be addressed as tumour cell embolism rather than metastasis. Since neither the mice nor the donor had evidence of lymphogenic or hematogenic metastasis, we conclude that time for metastasis exceeds the time limitation owed to local tumour growth. Whether this is due to the rapid growth rate or due to potentially little disposition of the cells to degrade extracellular matrix and therefore has a generally low tendency to metastasise remains unclear. The absence of bone metastasis is a remarkable fact, which is contrary to the high incidence of bone metastasis in canine and human patients [5,19]. Potentially there was not enough time for bone metastasis to develop due to rapid local tumour growth, on the other hand, this particular tumour might have a reduced tendency for skeletal metastasis. At first glance this reduces the value of the present tumour model for comparative oncology, but this interesting feature might be used for further studies focussing on skeletal metastasis in particular, if the cause for the absence of metastasis to the skeleton in this otherwise highly malignant prostate carcinoma is detected. The tendency to show necrotic areas in the centre might be due to rapid tumour growth.

The number of cells obviously did not seem not to have a direct impact on tumour growth, since all mice that had been inoculated with 1 × 10^5 ^cells developed tumours, but 50% of the animals that received 5 × 10^5 ^cells did not; although this is not considered to be statistically significant, it suggests that an even lower number of cells might have been sufficient to induce a tumour. The difference between male and female animals is not statistically significant.

The Ki67 indices in both, the original tumour and the experimentally induced tumours, were considered to be high. The difference between the original tumour on one hand and the subcutaneous and intraperitoneal masses on the other hand were statistically not significant. In human medicine a high Ki67 index has shown to be associated with a poor prognosis [20]. Considering the histological characteristics of the described tumour, the high Ki67 index is not a surprising finding. Comparison of the Ki67 index of the original tumour to the xenograft revealed no change in proliferation.

One limiting factor is that two of the nineteen mice in the study developed thymoma within the course of the study. This is a frequent finding in older NOD-Scid mice [16,17]. Therefore the animals must not be considered to be healthy.

## Conclusion

The canine prostate carcinoma cell line CT1258 demonstrated to be tumourigenic in the NOD-Scid mouse. Tumours induced in mice resembled the biological behaviour of the tumour from which the cell line was originally derived regarding growth pattern and histological appearance. Therefore we conclude that the use of this animal model will provide results with a high predictability towards clinical use in veterinary medicine and due to the correlation between canine and human prostate carcinoma in humans as well. The lack of skeletal metastasis is a potential field for further studies.

## Competing interests

The authors declare that they have no competing interests.

## Authors' contributions

MAMF was involved in study-design, implemented the in vivo tasks and the histopathology, and wrote the paper, HME was involved in study-design and coordination of in vitro work up, JTS and KAS assisted with dissection of the animals and in vitro work up, SaW prepared the cells for inoculation, SuW was involved in study-design, NR–B was involved in chromosomal preparation, cytogenetic analyses and karyotyping, MD and HHJH were involved in study-design and supervising the *in vivo* work up, JB and IN incited the study and coordinated the operational procedure. All authors read and approved the final manuscript.

## Pre-publication history

The pre-publication history for this paper can be accessed here:


